# Multifaceted Analysis of the Regional Landscape and Environmental Pollution of Industrial Categories and Key Enterprises

**DOI:** 10.3390/toxics14070574

**Published:** 2026-06-29

**Authors:** Hao Zhang, Bin Zhao, Yifei Liu, Hao Zheng, Yinan Song, Yang Yang, Xiaoyu Liu, Zhifeng Li, Jing Jiang

**Affiliations:** 1 Technical Centre for Soil, Agriculture and Rural Ecology and Environment, Ministry of Ecology and Environment, Beijing 100012, China; 2 Institute of Eco-Environmental and Soil Sciences, Guangdong Academy of Sciences, Guangzhou 510650, China; 3 Zhejiang Xingtuo Ecological Environment Co., Ltd., 733 Jianshesan Road, Hangzhou 311200, China; 4 CNPC Research Institute of Safety & Environment Technology, Beijing 102206, China

**Keywords:** environmental management, regional distribution, wastewater treatment, urban development, pollution emissions

## Abstract

Industrial emissions are a central environmental concern, particularly with respect to the spatial distribution of major enterprises and the identification of key determinants. Traditional research has largely focused on characterizing the current status of these enterprises, but this approach exhibits several notable shortcomings. These include a lack of regional statistical analysis, an absence of a comprehensive industrial typology, inadequate cross-evaluation of enterprise scale and pollution emissions, and insufficient exploration of socioeconomic correlations. We introduce a multifaceted evaluation framework for Key Environmental Supervision Units (KESUs), focusing on key industrial classifications and their underlying development drivers. The analysis utilized a comprehensive dataset covering 153,107 individual KESUs across six categories from 2020 to 2024, incorporating distribution patterns across 31 provincial-level regions, 28 industrial classifications of national economic activities, and 18 socioeconomic impact factors. The results showed that KESUs in East China accounted for 41.7% of the total, with the highest concentrations in industrialized cities and economically developed zones. Manufacturing was identified as the dominant industrial classification, with chemical raw materials and products comprising the largest subcategory (13.0% of total KESUs in 2024). Atmosphere KESUs and water KESUs represented the largest proportions, accounting for 29.7% and 25.3% of single-type KESUs, respectively. This study provides a systematic analysis of KESUs, offering a detailed mapping of distribution patterns, emission characteristics, and control challenges for major pollution sources. The findings can provide critical insights to support decision-making aimed at improving regional pollution source management and advancing environmental protection practices.

## 1. Introduction

**Figure 1:**
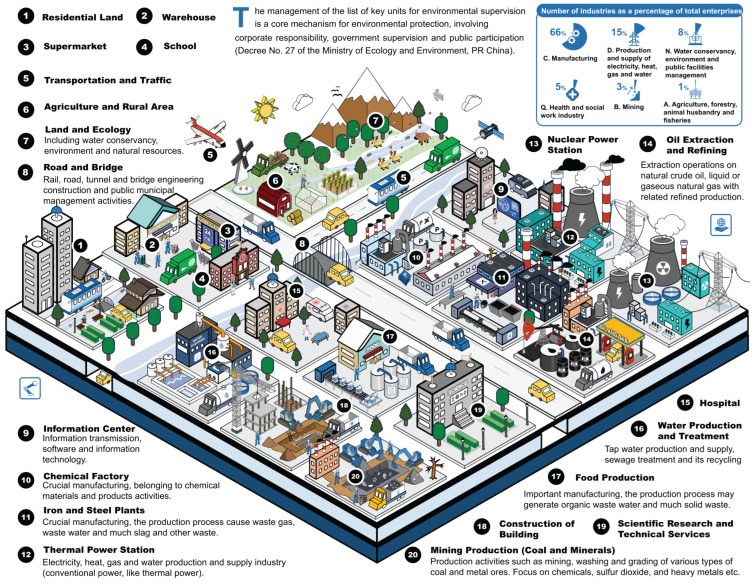
Schematic illustration of city development, key units, and pollution emissions. The detailed roles of each area and unit, as well as the proportions of important industrial types, are described according to industrial classification.

The accelerated pace of industrial development has intensified the challenge of balancing economic growth, industrial value-added, and environmental sustainability, a complex and systemic issue that continues to demand attention [1,2,3]. Factory emissions primarily affect the environment through wastewater discharge [4], exhaust gases [5], and solid waste [6], among other pollutants. Untreated effluents have been shown to adversely impact environmental media, such as water, air, and soil quality [7,8], thereby exacerbating ecological imbalances and increasing human health risks [9]. Source management for high-impact enterprises is not only critical for enhancing governance efficiency and reducing management costs but is also a central focus for global environmental regulators and remains a prominent area of scientific research [10,11]. The environmental characteristics of major human settlements during social development are closely linked to impact factors (Figure 1). Beyond conventional industrial and mining enterprises and energy-intensive polluting industries, institutions such as hospitals, research centers, and wastewater treatment facilities also warrant significant attention [12,13,14].

Among the various source control measures, the classification of enterprises into six categories of Key Environmental Supervision Units (KESUs), based on pollutant emission levels, toxic substances, environmental risk management needs, and improvement requirements, represents an innovative initiative and a critical institutional safeguard in China’s environmental management (Figure 1) [15,16]. These six categories can be further subdivided according to the environmental elements they affect, including water, air, soil, groundwater, noise, and environmental risk (Figure S1) [17]. Strengthening environmental management within KESUs provides an effective mechanism for addressing pollution at its source and ensuring compliance with environmental legislation, thereby shifting from reactive measures to proactive management [18,19,20,21].

Current scientific research on enterprise management is marked by an obvious lack of studies, with most existing literature focusing on policy interpretation and the application of remediation technologies [22,23,24,25]. There are several key research gaps that require attention, including analyses of industrial distribution patterns, investigations into the drivers of corporate behavior, and studies on regional collaborative management strategies [26,27,28,29,30]. A significant gap is the absence of systematic inventories of major enterprise pollution sources [31,32,33]. Furthermore, data on the evolution, spatial distribution, and primary pollutants of these sources remains critical for environmental management [34,35,36]. The role of socioeconomic factors in determining the number and distribution of industrial pollution sources remains an area of research that has not yet been extensively explored [37,38,39,40].

Hence, a multifaceted analytical framework was proposed to assess the distribution patterns and quantitative evolution of KESUs. Utilizing publicly available official data and authoritative sources, we mapped the regional locations of nearly 100,000 KESUs across China for multiple years, conducted detailed provincial-level analyses, and characterized the distribution features of key KESU types. This study examined KESU growth rates and spatial relationships across 31 provincial regions, identified key factors within 28 subcategories of six major industrial classifications, and assessed correlations with the emission characteristics of six key types of pollutants. A range of socioeconomic analyses were conducted, incorporating data on regional GDP and population density. The insights derived from this study are highly relevant for urban planning, environmental protection, and policy formulation.

In this work, we constructed a dataset comprising KESUs data information from various regions across different years. Utilizing geolocation techniques, cluster analysis, correlation analysis, proportional chord diagrams, and socioeconomic impact factor analysis, we examined the evolving trends of KESUs across distinct regions and industries and pinpointed the critical factors influencing the quantity and distribution of KESUs. This multidimensional framework, presented through visualization and structured analysis, can provide comprehensive performance evaluations and integrated solutions for pollution source risk control.

## 2. Methodology

### 2.1. Related Abbreviations

All regions in this study were referred to via the following standard abbreviations [19]: Beijing-BJ, Tianjin-TJ, Hebei-HE, Shanxi-SX, Inner Mongolia (also named Neimenggu-IM), Liaoning-LN, Jilin-JL, Heilongjiang-HL, Shanghai-SH, Jiangsu-JS, Zhejiang-ZJ, Anhui-AH, Fujian-FJ, Jiangxi-JX, Shandong-SD, Henan-HA, Hubei-HB, Hunan-HN, Guangdong-GD, Guangxi-GX, Hainan-HI, Chongqing-CQ, Sichuan-SC, Guizhou-GZ, Yunnan-YN, Tibet (also named Xizang-TB), Shaanxi-SN, Gansu-GS, Qinghai-QH, Ningxia-NX, Xinjiang-XJ, Hong Kong-HK, Macao-MO, and Taiwan-TW, and the XinJiang Production and Construction Corps-XPCC. Statistics for HK, MO, TW, and XPCC are not described in this study. The provincial-level central cities referred to in this article are typically the provincial capitals.

Different KESUs were divided into six types, such as: key water pollution discharge units (W-KESUs), key groundwater pollution prevention and control discharge units (G-KESUs), key air pollution discharge units (A-KESUs), key noise pollution discharge units (N-KESUs), key soil pollution supervision units (S-KESUs), and key environmental risk control units (R-KESUs).

### 2.2. Data Collection

Detailed resources and information on KESUs are available for each region in Table S1, and all data was collected from open and published source. We constructed a longitudinal panel dataset covering 393,806 unique KESUs over the 5-year observation period from 2020 to 2024. The annual sample size exhibited a steady increase, with 54,866 units documented in 2020, 68,942 in 2021, 83,502 in 2022, 91,426 in 2023, and 95,070 in 2024. Notably, data entries across this time interval were derived from standardized regulatory reporting systems, yielding far more complete and detailed information than datasets collected in earlier years. The curated dataset systematically archived critical unit-level characteristics, including precise geographic coordinates, categorized industrial-scale metrics, regulatory jurisdiction-based classification labels, and supplementary administrative attributes. It is important to note that a single enterprise or institution may be classified under multiple KESU categories concurrently. Pollution emissions of KESUs were available at https://permit.mee.gov.cn/ (31 December 2024). Statistics of the socioeconomic factors and total emissions were available at https://data.stats.gov.cn/ (31 December 2024).

### 2.3. Analysis Framework

The research approach involved statistical analysis of the collected data such as the quantity and distribution of KESUs, combined with regional and industry characteristics for feature characterization and analysis. It also incorporated socioeconomic impact factors to identify key information affecting the classification and grading of KESUs across different regions.

Location and number of KESUs. The coordinates of KESUs were marked on the map using ArcMap 10.8 software, and the number of KESUs was counted at the city scale to provide greater statistical value. Through the filtering mechanism of Excel 2021 software, we optimized the KESUs that appeared repeatedly each year from a total of 393,806, resulting in a total of 153,107 KESUs over a span of 5 years.

Cluster analysis. It was generated based on quantitative data, and a dendrogram in polar coordinates was used to show the affiliation of different classes. In this work, the different regional values of KESUs were normalized to understand which could be clustered into a class at a visual level during the period from 2020 to 2024. The cluster polar heat map dendrograms were generated using OriginPro 2022 with the defined parameters of cluster method (group average) and distance type (squared Euclidean) and were classified into resident and industrial uses.

Correlation analysis. Correlation analysis was used to measure how closely different 18 socioeconomic impact factors influenced the number of KESUs, with Spearman correlation coefficient being used due to the independent feature of the distribution of data and its insensitivity to outliers. Correlation plots were produced using OriginPro 2022 with the Spearman correlation type, excluding missing values pairwise. Significant levels were set at 0.05, 0.01, and 0.001, respectively.

## 3. Results and Discussion

### 3.1. Spatial Distribution and Numerical Focus of KESUs

**Figure 2:**
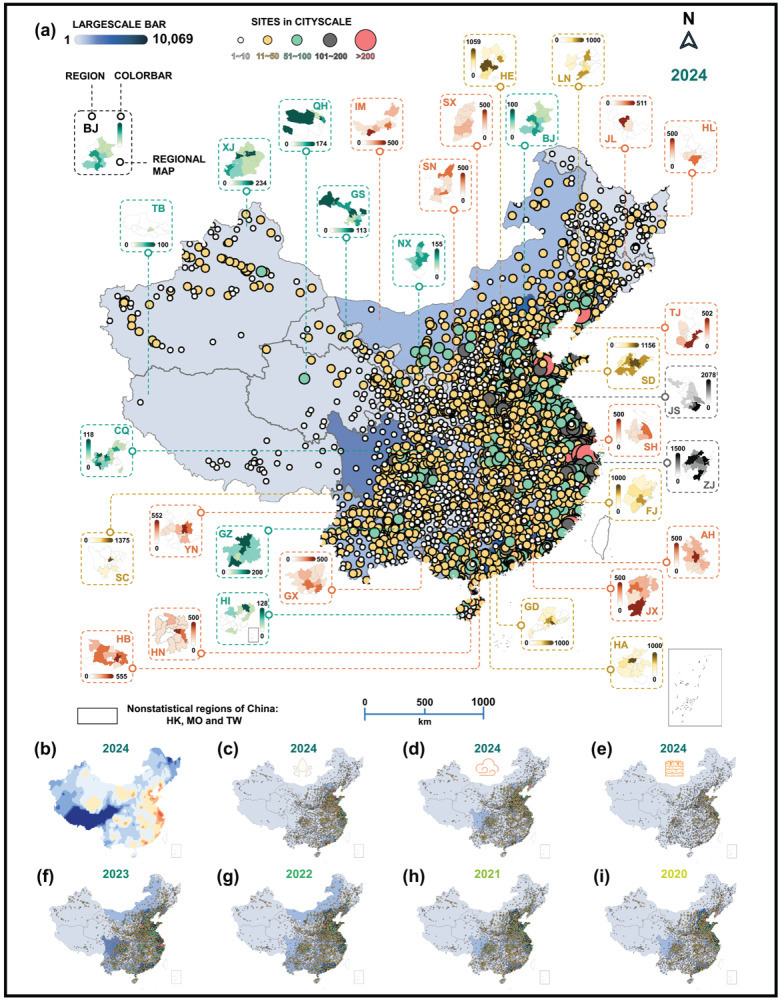
Location and distribution of KESUs in different regions. (**a**) Map of KESUs in 2024. The detailed distribution of KESUs at the provincial scale is shown in the inset. (**b**) Fit distribution heat maps of KESUs in 2024. (**c**) Map of W-KESUs in 2024. (**d**) Map of A-KESUs in 2024. (**e**) Map of S-KESUs in 2024. (**f**) Map of KESUs in 2023. (**g**) Map of KESUs in 2022. (**h**) Map of KESUs in 2021. (**i**) Map of KESUs in 2020.

Figure 2 shows the distribution of KESUs by type across different years. It displayed the regional- and provincial-level distribution of KESUs in China for 2024 (Figure 2a). The data were classified at the county-level urban area scale, categorizing KESUs into five tiers based on quantity, followed by 1~10, 11~50, 51~100, 101~200, and >200, with corresponding data points plotted on the map. It indicated that KESUs were located in most cities nationwide, with a concentration in industrially robust and economically developed regions, such as the Beijing–Tianjin–Hebei area, the East China coastal zone, the Yangtze River Delta, South China, and the Sichuan–Chongqing region [41,42,43,44]. Notably, distinct patterns emerged in the regional distribution of KESUs. In the western regions, KESUs tended to cluster around core cities, whereas in the eastern regions, a more dispersed and balanced distribution was observed within provincial boundaries.

Cities with more than 200 KESUs were predominantly focused on the eastern coastal regions, as illustrated by the heatmap (Figure 2b). A statistical analysis of KESU counted by tier for 2024 indicated that the majority of cities (55.3%) host between 11 and 50 KESUs, with 23.7% of cities falling within the 11~20 range. Cities with fewer than 50 KESUs represented 83.7% of the total, while those with more than 100 KESUs accounted for only 4.8%. It suggested that the number of resource-intensive KESUs is relatively small, with most cities containing fewer polluting enterprises. This distribution indicates favorable trends in environmental governance, industrial restructuring, and policy regulation within these regions [45,46,47].

The distribution of W-KESUs, A-KESUs, and S-KESUs across the three environmental factors in 2024 exhibited the presence of all three types of KESUs in most regions, with densities following the order A-KESUs > W-KESUs > S-KESUs (Figure 2c–e). A-KESUs were predominantly located in northern regions, with higher densities observed in economically developed mountainous and basin areas, such as the Sichuan–Chongqing region [48,49]. In contrast, in water-rich regions such as the Yangtze River Delta [50,51], W-KESUs exhibited a higher density relative to A-KESUs.

**Figure 3:**
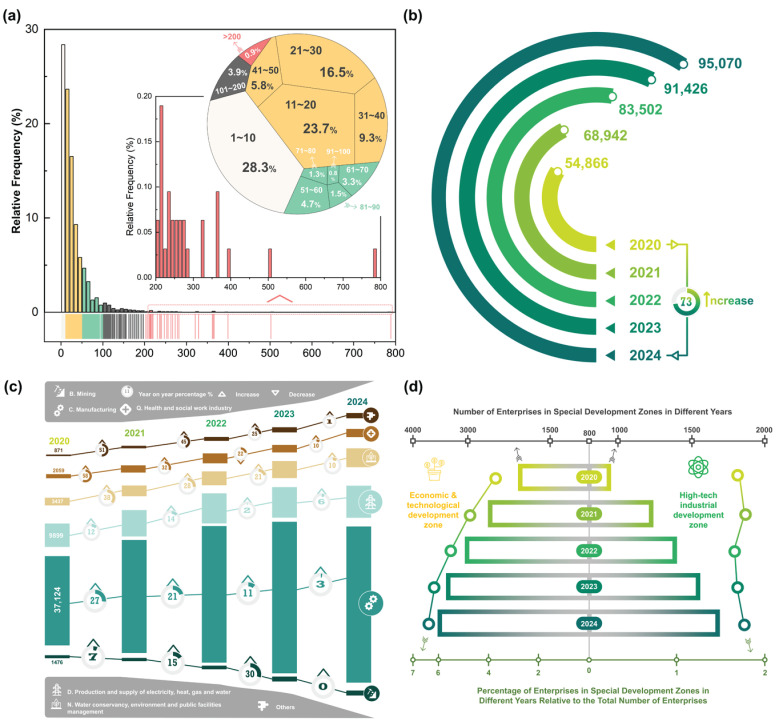
(**a**) Proportion KESUs in different regions (2024); (**b**) Statistics on the number of KESUs over different years; (**c**) Evolution of the KESUs over different years, with the proportions of diverse industrial classifications; (**d**) Number of KESUs in the economic and technological development zone and high-tech industrial development zone.

A longitudinal analysis of national KESU distributions from different years showed a gradual increase in KESU density, with particularly marked growth in Central China, IM, and the SC-CQ regions (Figure 2f–i). This trend was reflective of the ongoing tightening of ecological and environmental protection policies, coupled with the refinement of environmental management practices [52,53]. Figure 3b illustrated a steady increase in the number of KESUs over time, with a notable growth rate of 73.3% from 2020 (54,866 KESUs) to 2024 (95,070 KESUs). The KESUs were classified according to national economic sectors, and the analysis focused on six major categories: (1) mining, (2) manufacturing, (3) production and supply of electricity, heat, gas, and water, (4) water conservancy, environment and public facilities management, (5) health and social work, and (6) others. The analysis showed an overall annual growth trend across the different sectors, with the highest increase observed in 2021. It indicated a consistent decline in the growth rate of KESUs in the manufacturing sector and primary functional industries, which collectively represented a substantial proportion of the economy.

As key indicators of urban development, high-tech zones prioritized industries such as high-tech and strategic emerging sectors, while economic development zones emphasized the enhancement of traditional industries and regional economic coordination [54,55]. Figure 3d showed the total number of KESUs and their proportion within high-tech zones and economic development zones over time. It shows a consistent annual increase in the number of KESUs in both zones. A significantly larger number of KESUs were consistently hosted by the economic development zones in comparison to the high-tech zones. The proportion of KESUs in economic development zones relative to the national total had gradually increased, while the proportion in high-tech zones has remained relatively stable.

A detailed analysis of the cumulative 153,107 KESUs over the five-year period (Figure S2 and S3) shows that the majority (36.1%) of KESUs were observed only once during the statistical period, while 21.1% were recorded on two separate occasions. This finding indicated that, owing to the dynamic updating mechanism, some KESUs were eliminated from the dataset. It was presumably attributable to factors such as reduced emissions, industrial restructuring, or alterations in local environmental carrying capacity (Figure S2). Most units (approximately 70%) were classified under a single KESU category (Figure S3). Among these, A-KESUs accounted for 29.7% of the total, while W-KESUs represented 25.3%. Enterprises classified under multiple KESU categories were primarily categorized in two combinations, with 4.1% of them classified as both A-KESUs and W-KESUs.

### 3.2. Regional Management of KESUs

**Figure 4:**
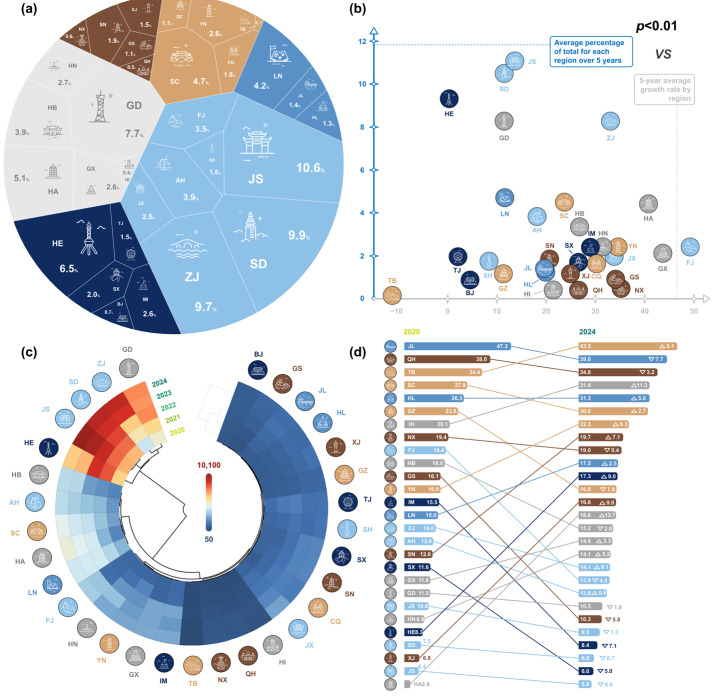
(**a**) Percentage of KESUs by region in 2024. (**b**) Relationship between the average percentage of total KESUs for each region over 5 years and the 5-year average growth rate of regional KESUs. (**c**) Cluster analysis of KESU counts across different years and regions. (**d**) Changes in the percentage of KESUs in provincial capitals relative to their regional totals between 2020 and 2024.

Figure 4 illustrates the number, scale, and developmental trends of KESUs across different regions (2024). The East China region accounted for the largest proportion of KESUs, exceeding 40% (41.7%), which can be attributed to its significant industrial scale and rapid economic growth (Figure 4a). Within this region, the provinces of JS, SD, and ZJ emerged as the top three, accounting for 10.6%, 9.9%, and 9.7%, respectively. In contrast, KESU proportions in Northeast (NE) and Northwest China (N) were notably lower, at 6.9% and 5.6%, respectively, highlighting substantial regional disparities. HE and LN lead in KESU numbers within North China (N) and Northeast China (NE), respectively. A correlation analysis between the five-year average growth rates and the five-year average shares across regions showed that areas with a higher quantitative share tend to exhibit lower growth rates (Figure 4b). The majority of regions showed an average share of less than 5.0% over the five-year period, with growth rates ranging from 10.0% to 40.0%. FJ recorded the most substantial growth rate at 49.5%, while TB registered a negative average growth rate, indicating a gradual decline in KESUs. The linear and polynomial fitting results for regional data were not statistically significant (R^2^ < 0.1); however, provinces with more robust economic development exhibited notable differences and clustering patterns compared to other regions.

Cluster analysis of regional distributions over the years indicated that areas with a higher number of KESUs exhibited similar temporal patterns, whereas regions with fewer KESUs showed no discernible similarity (Figure 4c). A comparison of identical regions across different years indicated that the years 2021 and 2022, as well as 2023 and 2024, exhibited similar trends, whereas 2020 stands out as an anomaly. The proportion of KESUs in central cities across different regions also demonstrated notable shifts over the five-year period (Figure 4d). The central cities of HA experienced the highest increase, with a 13.7% rise, while that of JL central cities showed the largest decline, at 8.1%. Notably, certain cities with high proportions, such as JL, QH, and TB, exhibited minimal changes in their rankings over the five years. In contrast, the central city of TB experienced relatively high growth, with a rate of 9.1%. The fluctuations in KESUs in central cities were often reflective of industrial restructuring and upgrading processes, while higher growth rates were typically indicative of stronger resource agglomeration effects within the region [56,57].

### 3.3. Impact on Industrial Categories of KESUs

**Figure 5:**
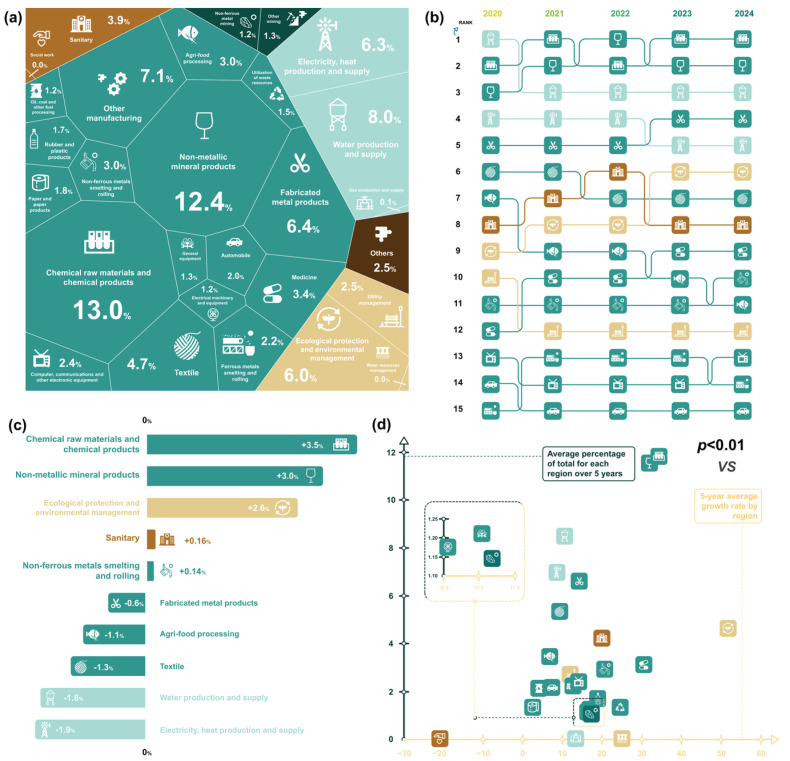
Number and proportion of KESUs across different years and industries. (**a**) Proportion of KESUs by industrial category in 2024. (**b**) Changes in industrial categories over the 5-Year Period 2020–2024. (**c**) Changes in the proportion of KESUs across major industries between 2024 and 2020. (**d**) Relationship between the 5-year average proportion of total KESUs for each region and the 5-year average growth rate.

The analysis of KESUs by industrial classification across different years was presented in Figure 5 and Figure S4. Industrial classification follows the principles outlined in the Industrial Classification for National Economic Activities (GB/T 4754-2017) [58]. The manufacturing sector accounted for the largest proportion of KESUs, representing 68.2% of the total (2024) (Figure 5a). The chemical raw materials and chemical products and non-metallic mineral products sectors ranked first and second, with proportions of 13.0% and 12.4%, respectively. A comparison of KESU proportions over time revealed a general shift within the manufacturing sector, moving from a more dispersed distribution to a greater concentration in these two high-proportion industries. Additionally, the proportion of KESUs in the “Production and Supply of Electricity, Heat, Gas, and Water” category declined annually (Figure S4).

Analysis of the industry rankings over time showed no significant shifts, with rankings remaining relatively stable between 2021 and 2022, and between 2023 and 2024. As depicted in Figure 5b, the top three industrial classifications—production and supply of water, chemical raw materials and chemical products, and non-metallic mineral products—consistently maintained their positions. The percentage change in KESUs between 2024 and 2020 revealed that the chemical raw materials and chemical products category experienced the most significant increase, rising by 3.5%, followed by non-metallic mineral products, which increased by 3.0% (Figure 5c). Certain sectors, such as those involved in the production of sanitary products and the smelting and rolling of non-ferrous metals, showed no significant changes. The production and supply of electricity, heat, gas, and water sectors exhibited a marked decline, reflecting ongoing industrial restructuring and a gradual reduction in the proportion of high-energy-consuming industries [59,60,61].

A correlation analysis between the five-year average growth rates and average shares across industry categories indicated that the sectors with higher KESU proportions tended to exhibit higher growth rates. Figure 5d shows trends across industry categories that differed from the regional trends. At the 0.01 significance level, the mean population differed significantly from that of KESUs across different industrial categories. The mean growth rate for most industries ranged from 0% to 30%, with ecological protection and environmental management showing the largest increase at 51.6%. Conversely, the Social Work sector experienced a significant decline, with a decrease of 20.8%. The fitting of confidence intervals or uncertainty estimates for the average number of KESUs and their growth rate variations across different industries demonstrated a good linear fit (Figure S5). These shifts in KESU numbers signal the gradual phase-out of traditional, high-energy-consuming, and high-emission industries. Furthermore, environmental management efforts were increasingly focused on high-pollution-risk sectors, with emerging environmental protection enterprises becoming the primary targets of regulatory attention [62,63].

### 3.4. Diverse Types of KESUs

**Figure 6:**
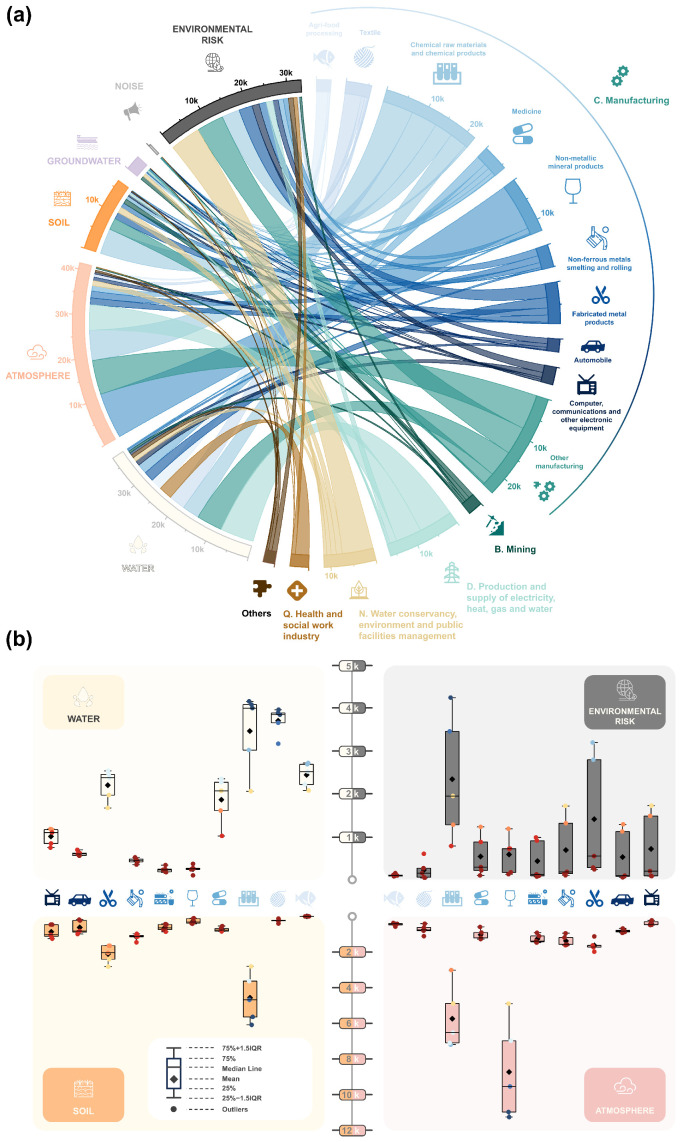
Quantities and interrelationships among six types of KESUs across different years and industries. (**a**) Flow relationships between major industries and six types of KESUs in 2024. (**b**) Statistical relationships between key manufacturing sectors and four categories of KESUs across different years.

**Figure 7:**
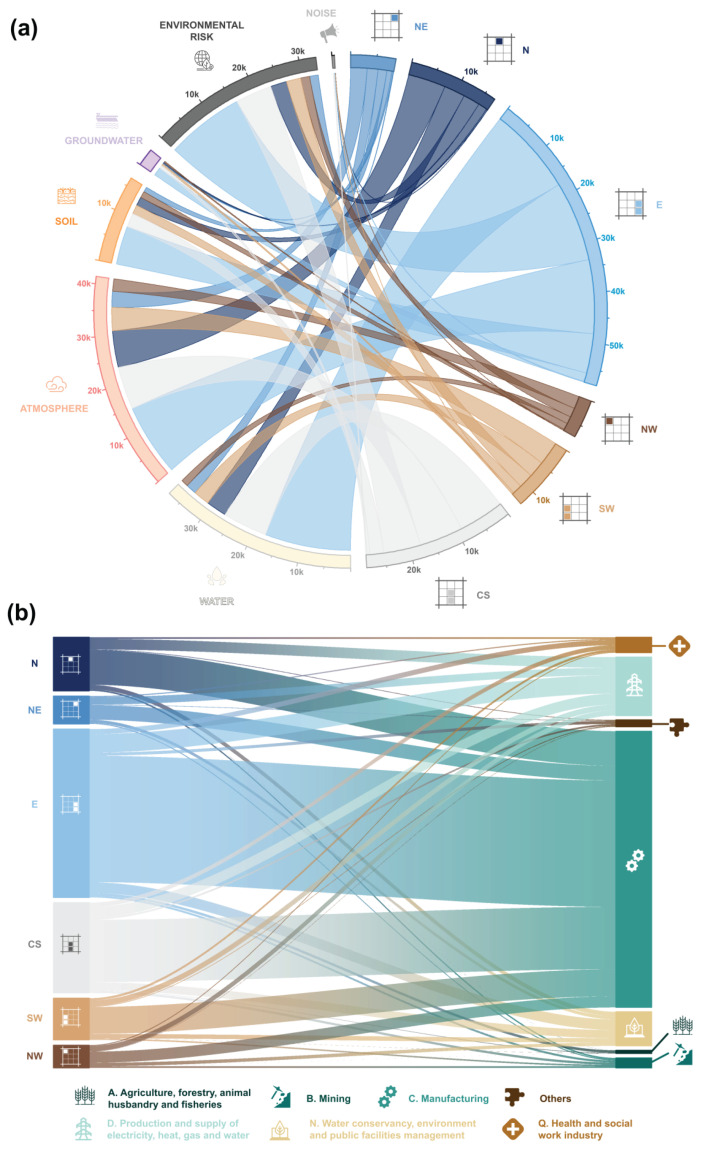
Quantities and interrelationships among six types of KESUs across different years and industries. (**a**) Flow relationships between different regions and six types of KESUs in 2024. (**b**) Interrelationships between different regions and industries in 2024.

Figure 6 and Figure 7 present an analysis of the regional and industrial characteristics of different KESU types. The association between the six KESU categories (W-KESUs, A-KESUs, S-KESUs, G-KESUs, R-KESUs, and N-KESUs) and various industry types, along with their quantitative dynamics (2024), is depicted in Figure 6a. A-KESUs and W-KESUs were the most prevalent, representing 39.5% and 43.5% of the sample, respectively, while N-KESUs were the least numerous, accounting for just 0.5%. Notably, A-KESUs were predominantly concentrated in three industries: non-metallic mineral products (26.5%), other manufacturing (19.8%), and chemical raw materials and chemical products (17.4%). In contrast, W-KESUs exhibited a more balanced distribution across industries compared to A-KESUs.

An analysis of five-year statistics for the four KESU types across major manufacturing sectors revealed distinct numerical distributions for each KESU category. The data indicated significant variations. For W-KESUs, the textile and chemical raw materials and chemical products sectors exhibited relatively stable annual distributions. In contrast, R-KESUs demonstrated considerable fluctuations in their contributions to the same industry across different years. A-KESUs and S-KESUs followed analogous patterns, although non-metallic mineral products showed a marked influence on A-KESUs, exhibiting considerable variation across years, alongside chemical raw materials and chemical products. The number and bidirectional relationships of the six KESU categories across different regions are presented in Figure 7a (2024). In northern regions (N, NE, NW), A-KESUs accounted for a higher proportion of the total KESUs, suggesting a stronger emphasis on atmospheric environmental management. A comparative analysis of the six KESU categories revealed that East China (E) contributed the most, followed by South China (CS). These two regions accounted for 70.7% of W-KESUs and 70.3% of R-KESUs.

Figure 7b illustrates the correlation analysis across industries and regions (2024). With 45.8% of all KESUs within the m manufacturing category, the East China (E) was a key player in the global supply chain. In contrast, Northeast China (NE) showed a reduced disparity between manufacturing and the “Production and Supply of Electricity, Heat, Gas, and Water” sectors (52.4% vs. 26.7%). In terms of water conservancy, environmental protection, and public facilities management, the distribution of KESUs across regions was relatively balanced, with no significant disparities observed.

### 3.5. Pollution Emissions in Key Regions

**Figure 8:**
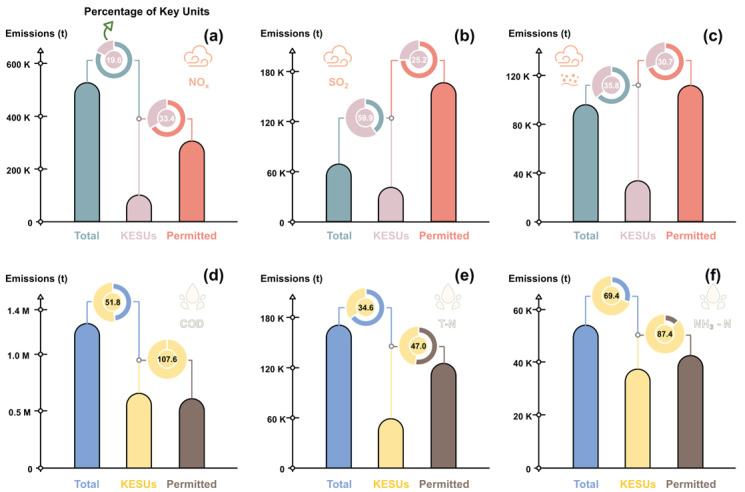
Water environment and major air pollutant emissions in focus regions. Regional total, A-KESUs, and permitted air pollutant emissions: (**a**) NO_x_; (**b**) SO_2_; (**c**) particulate matter. Regional total, W-KESUs, and permitted water pollutant emissions: (**d**) COD; (**e**) total nitrogen (T-N); (**f**) ammonia nitrogen (NH_3_-N).

The identification of key regions and industries was critical when analyzing the pollution emissions of KESUs. Based on the findings presented in Section 3.2 and Section 3.4, JS exhibits the highest proportion of KESUs among all regions. Given its substantial sample size, comprehensive coverage of industry types, and complete pollution emission data, we selected JS as the focal region to investigate the correlation between pollution emissions and industrial categories. The dataset included pollution emissions from 3704 A-KESUs and 4479 W-KESUs, representing a total of 6545 KESUs in JS during 2023 (Figure 8). The air pollution indicators analyzed were nitrogen oxides (NO_x_), sulfur dioxide (SO_2_), and particulate matter, while the water pollution indicators included chemical oxygen demand (COD), total nitrogen (T-N), and ammonia nitrogen (NH_3_-N) (Figure 8a–f) [63,64]. The emissions from these KESUs constituted a significant proportion of the region’s total pollution. Specifically, SO_2_, COD, and NH_3_-N emissions contributed to over 50% of the total, with contributions of 59.9%, 51.8%, and 69.4%, respectively. Notably, with the exception of COD, the pollutant emissions from KESUs were substantially below the permitted discharge limits. The results suggested that both W-KESUs and A-KESUs had effectively implemented environmental management and pollution control measures, meeting or exceeding regulatory standards. The discharge of COD, however, remained a key concern, as it was a high-emission pollutant (660,000 tons). When compared to W-KESUs, A-KESUs’ emissions of the three primary pollutants were consistently below their permitted limits.

**Figure 9:**
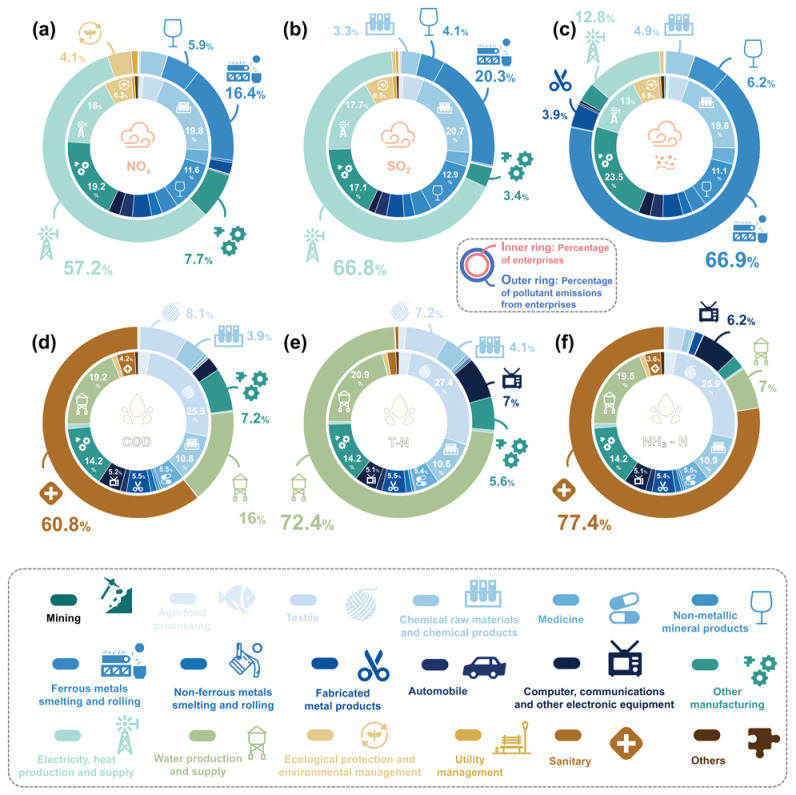
Analysis of the proportion of A-KESUs by industry sector and of their share of air pollutant emissions: (**a**) NO_x_; (**b**) SO_2_; (**c**) particulate matter. Analysis of the proportion of W-KESUs by industry sector and their share of water pollutant emissions: (**d**) COD; (**e**) total nitrogen (T-N); (**f**) ammonia nitrogen (NH_3_-N).

By deconstructing the proportion of KESUs and analyzing pollutant emissions, the relationships between different industries and their environmental impacts were investigated (Figure 9). Significant variations in air and water pollutant emissions were observed across industry sectors. The primary production and metal smelting sectors of A-KESUs were identified as major contributors to atmospheric pollution. The electricity and heat production sector accounted for 57.2% of NO_x_ emissions and 66.8% of SO_2_ emissions. Similarly, the water production and supply category, representing 20.9% of W-KESUs, contributed the majority of total nitrogen emissions (72.4%). In contrast, the ferrous metals smelting and rolling sector, though accounting for a smaller proportion of total KESUs (3.3%), was responsible for the majority of particulate matter emissions (66.9%). The sanitary wastewater sector, despite comprising less than 5% of the total KESUs, was the dominant contributor to COD and ammonia nitrogen emissions, accounting for 60.8% and 77.4%, respectively. These findings highlight the necessity of implementing targeted environmental management strategies in key developed regions. Specifically, atmospheric pollution control efforts ought to concentrate on high-energy-consuming industries, whereas water pollution management should give priority to specific sources with high emissions [65,66,67].

### 3.6. Socioeconomic Impact Analysis

The analysis of key factors influencing KESU management was inherently complex, as the outcomes were rarely driven by a single factor. A total of 18 socioeconomic indicators were employed to identify variables significantly impacting the five-year average quantity and total volume of KESUs (see Figure S6). These critical factors included industrial economic strength, enterprise and population dynamics, administrative jurisdictions, and other aspects that contribute to a nation’s economic vitality and social stability [14,19,68]. These indicators served as essential references for understanding the interrelations between environmental protection, KESUs dynamics, and broader macro-environmental influences.

The identified factors can be categorized into four broad groups: industrial indicators (e.g., regional GDP), social indicators (e.g., number of legal entities, employed population), fiscal indicators (e.g., budget revenue, disposable income of residents), and other indicators (e.g., total grain output). Spearman’s correlation coefficients were calculated to assess the relationships among these influencing factors. The analysis revealed a strong and statistically significant positive correlation between all categories of socioeconomic factors and both the annual average number and total volume of KESUs. It suggested that KESUs tend to increase in tandem with industrial development and economic growth [69,70]. It is worth noting that the positive influence of total and urban disposable income on KESU numbers was relatively modest. Further analysis of the socioeconomic impact on regional central cities largely mirrored the findings at the broader regional level (Figure S7). The positive influence of primary industry value-added on KESU numbers was found to be less pronounced than that of other factors [71,72].

### 3.7. Global Relevance and Future Work

Although this study primarily focused on regions and key economic development areas in China, it provides valuable insights for identifying key industries and managing emissions of critical pollutants, particularly in rapidly industrializing nations and mature manufacturing powerhouses. Through targeted analysis of various types of KESUs and identification of the core factors influencing corporate pollution emissions, coupled with the reinforcement of zoning and tiered management for industrial enterprises, we can incentivize enterprises to obtain and adhere to pollution discharge permits, thereby continuously enhancing pollution control and environmental management standards. This approach also serves as a catalyst for the treatment of key pollutants and global carbon mitigation efforts.

Future research endeavors will shift toward micro-level analyses, such as expanding the scope of pollutant emission analysis and incorporating additional pollutant emission screening criteria, as well as macro-level strategies, including efficient pollution control measures, reduction in pollutant emissions, and area-specific assistance and guidance. In addition, advanced deep learning techniques, including machine learning and neural networks, can be employed to conduct predictive analysis of KESUs for specific years and industries. Furthermore, fostering international collaboration, integrating environmental management practices from industrial enterprises in more developed countries, and addressing the emissions of emerging characteristic pollutants represent cutting-edge research avenues for scholars.

## 4. Conclusions

In summary, between 2020 and 2024, the number of KESUs increased from over 50,000 to more than 90,000, reflecting a growth of over 70%. Since the implementation of the policy, more than 150,000 enterprises have been included in the KESU list, with 66% of these operating within the manufacturing sector. The proportion of KESUs in economic development zones has steadily improved, while those in high-tech zones have remained relatively stable. The distribution of KESUs across sectors has exhibited considerable annual variation. Notably, the chemical raw materials and chemical products sector has consistently ranked among the top two. It reached 13.0% and increased by 3.5% over five years. Meanwhile, the electricity and heat production sector experienced a gradual decline, with a 1.9% decrease during the same period. Geographically, the East China and South China regions, characterized by diverse industrial structures and relatively advanced economies, hosted the highest density and largest number of KESUs. Consequently, these regions faced more substantial challenges related to pollution discharge and environmental management pressures. The primary KESU categories, A-KESUs and W-KESUs, accounted for 29.7% and 25.3%, respectively, of single-type KESUs. Notably, sanitary W-KESUs and electricity, heat production, and supply A-KESUs have been identified as major sources of water and air pollution, respectively. It revealed a significant positive correlation between KESU numbers and key socioeconomic factors, including regional GDP.

This study indicates that environmental protection policies are progressively reinforcing the supervision and administration of KESUs under environmental regulation. These involve major emitting enterprises subject to continuous monitoring, key industries under ecological and environmental oversight, and entities necessitating scientific management of environmental risks. Priority is given to critical domains such as water and air quality. The findings can offer significant methodological insights for precisely identifying industrial enterprises characterized by substantial pollutant discharge, elevated environmental risks, and heightened societal concern, thereby facilitating refined scientific environmental management and the implementation of targeted policies.

## Data Availability

The original contributions presented in this study are included in the article/Supplementary Material. Further inquiries can be directed to the corresponding authors.

## Supplementary Materials

The following supporting information can be downloaded at: https://www.mdpi.com/article/10.3390/toxics14070574/s1. Figure S1: Principles for developing different types of KESUs. Figure S2: Occurrence frequency of different KESUs during the period 2020~2024. Figure S3: Frequency of different types of KESUs between 2020 and 2024. Figure S4: Number and proportion of KESUs across different years and industries. Figure S5 Statistics of the relationship between different industrial classification with confidence intervals. Figure S6: Correlation analysis of 18 socio-economic impact factors in influencing the number of KESUs. Figure S7: Correlation analysis of 7 socio-economic impact factors in the city scale. Table S1: Related websites of the resources. References [17,58] are cited in Supplementary Materials.
