# Supplementary material for: Multifaceted Analysis of the Regional Landscape and Environmental Pollution of Industrial Categories and Key Enterprises
Source: Toxics. 2026 Jun 29;14(7):574. doi: 10.3390/toxics14070574 (PMC13418578; doi:10.3390/toxics14070574)
Supplement: Supplementary file 1 [file toxics-14-00574-s001.zip › toxics-4377970-supplementary.pdf]

Multifaceted Analysis on the Regional Landscape and Environmental Pollution of Industrial Categories and Key Enterprises

Hao Zhang [a,] *, Bin Zhao [b], Yifei Liu [a], Hao Zheng [c], Yinan Song [d], Yang Yang [a],

Xiaoyu Liu [a,] *, Zhifeng Li [b], Jing Jiang [a,] *

a. Technical Centre for Soil, Agriculture and Rural Ecology and Environment, Ministry of Ecology and Environment, Beijing 100012, PR China.

b. Institute of Eco-environmental and Soil Sciences, Guangdong Academy of Sciences, Guangzhou 510650, PR China.

c. Zhejiang Xingtuo Ecological Environment Co., Ltd, 733 Jianshesan Road, hangzhou 311200, PR China.

d. CNPC Research Institute of Safety & Environment Technology, Beijing 102206, PR China.

Figure S1 Principles for developing different types of KESUs.
Figure S2 Occurrence frequency of different KESUs during the period 2020~2024.
Figure S3 Frequency of different types of KESUs between 2020 and 2024.
Figure S4 Number and proportion of KESUs across different years and industries.
Figure S5 Statistics of the relationship between different industrial classification with confidence intervals.
Figure S6 Correlation analysis of 18 socio-economic impact factors in influencing the number of KESUs.
Figure S7 Correlation analysis of 7 socio-economic impact factors in the city scale.
Table S1 Related websites of the resources.
Reference

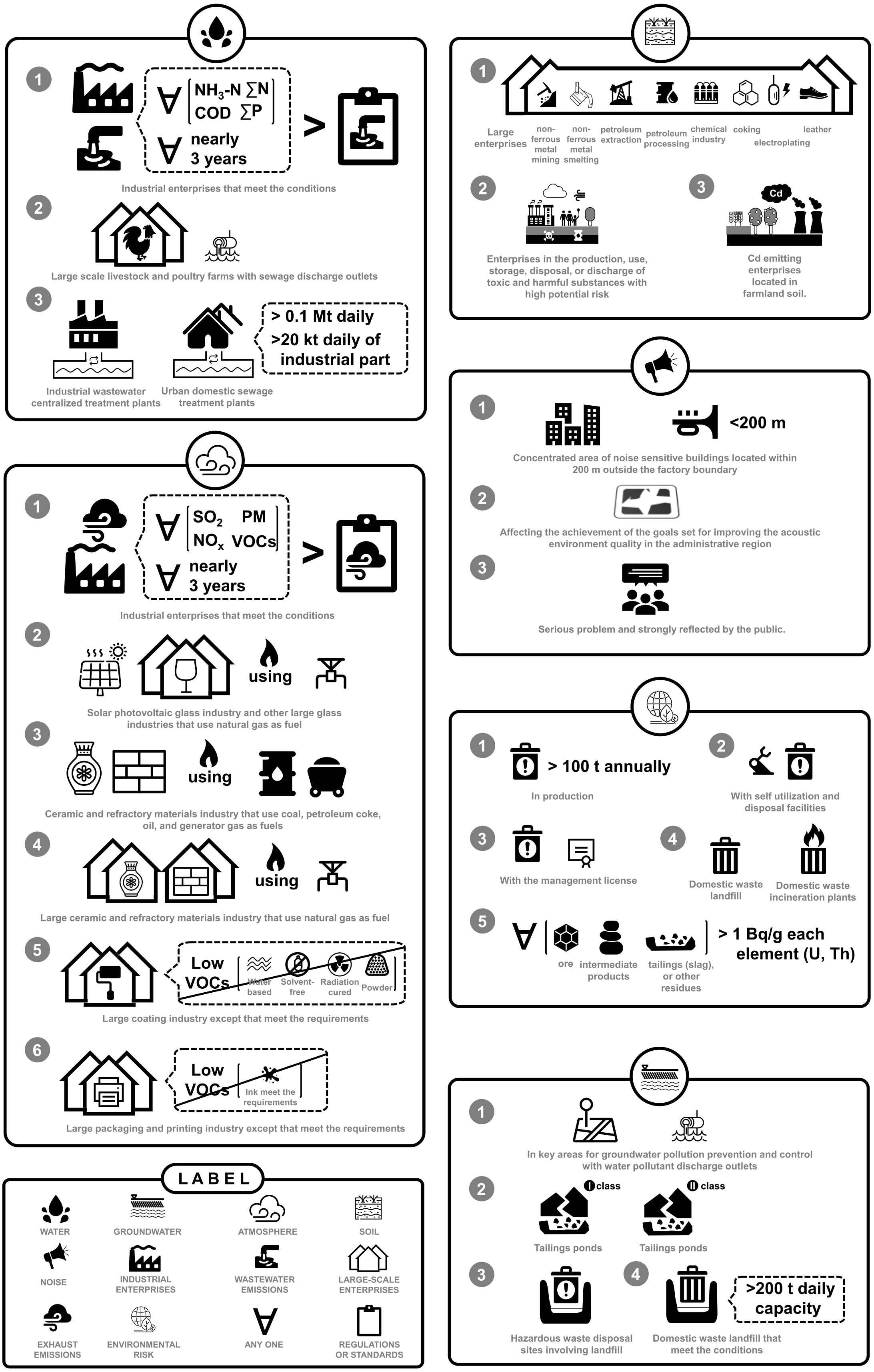

Figure S1 Principles for developing different types of KESUs[1-2].

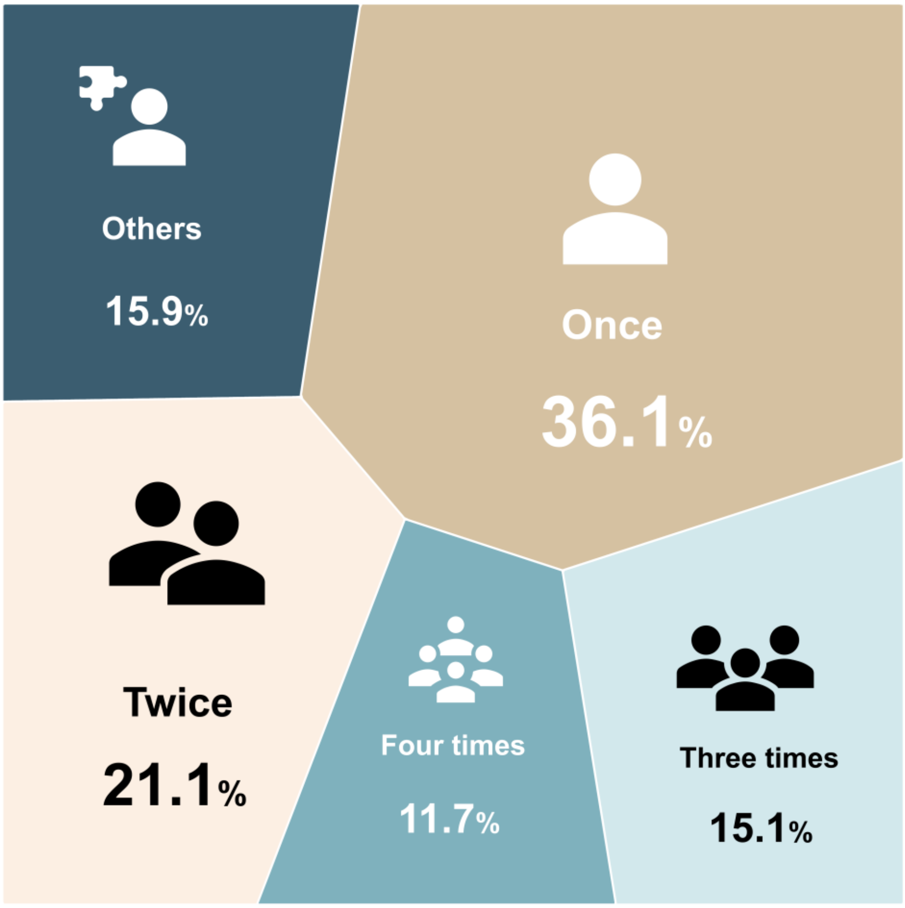

Figure S2 Occurrence frequency of different KESUs during the period 2020~2024.

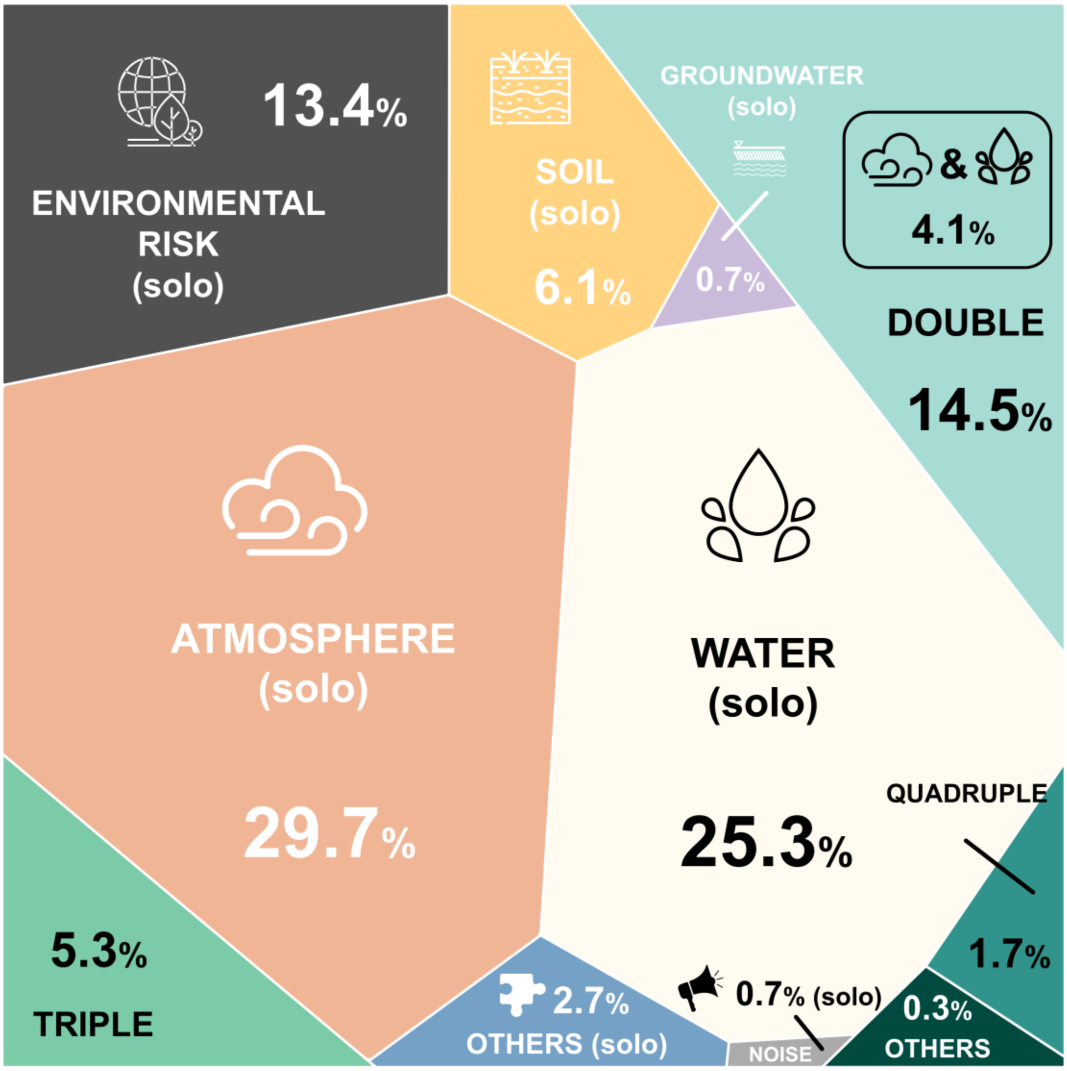

Figure S3 Frequency of different types of KESUs between 2020 and 2024.

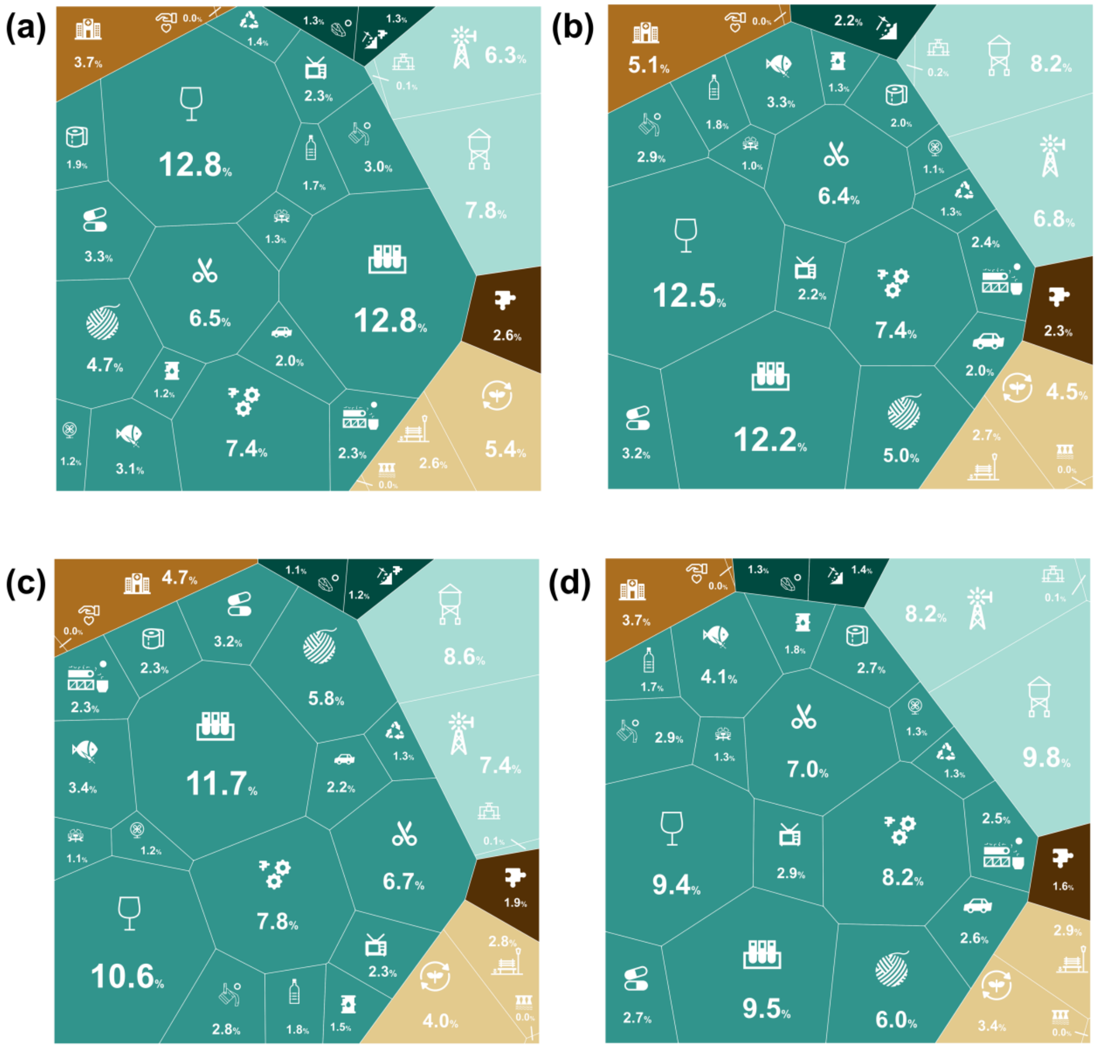

Figure S4 Number and proportion of KESUs across different years and industries. Proportion of KESUs by industry in 2023 (a); 2022 (b); 2021 (c); 2020 (d).

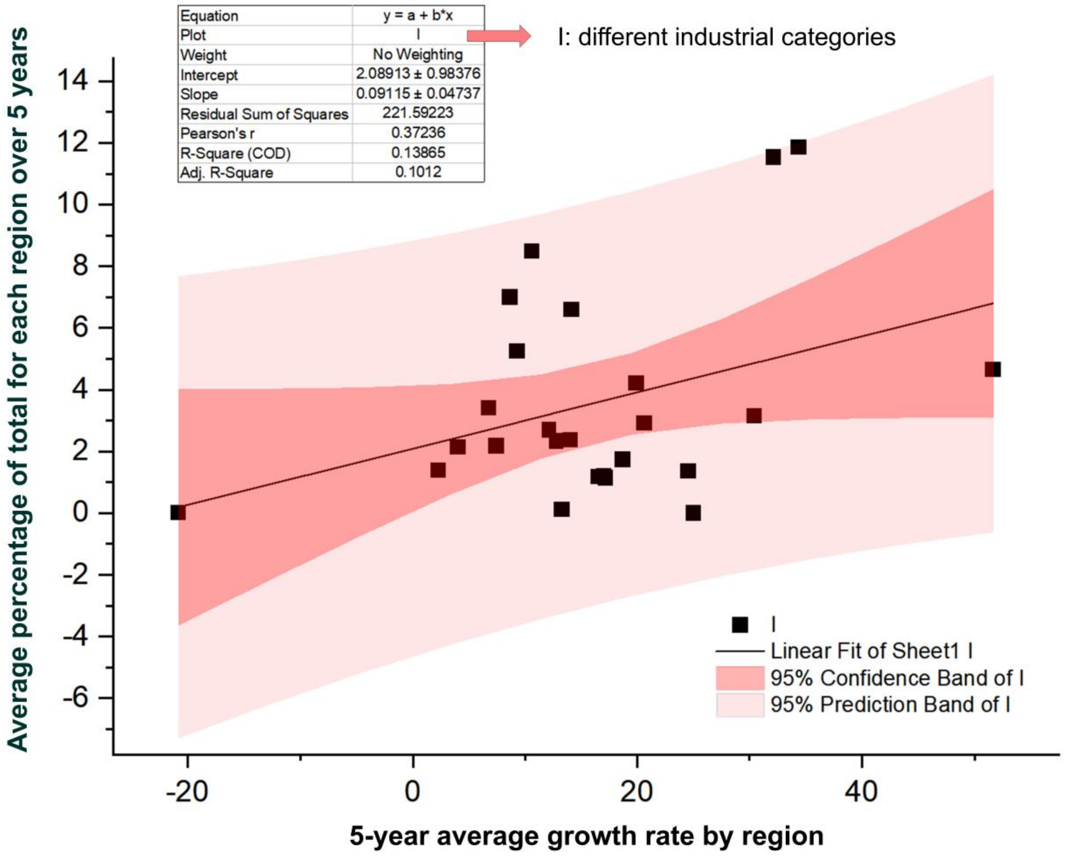

Figure S5 Statistics of the relationship between the average percentage of total for each region over 5 years and the 5-year average growth rate by industrial classification with confidence intervals.

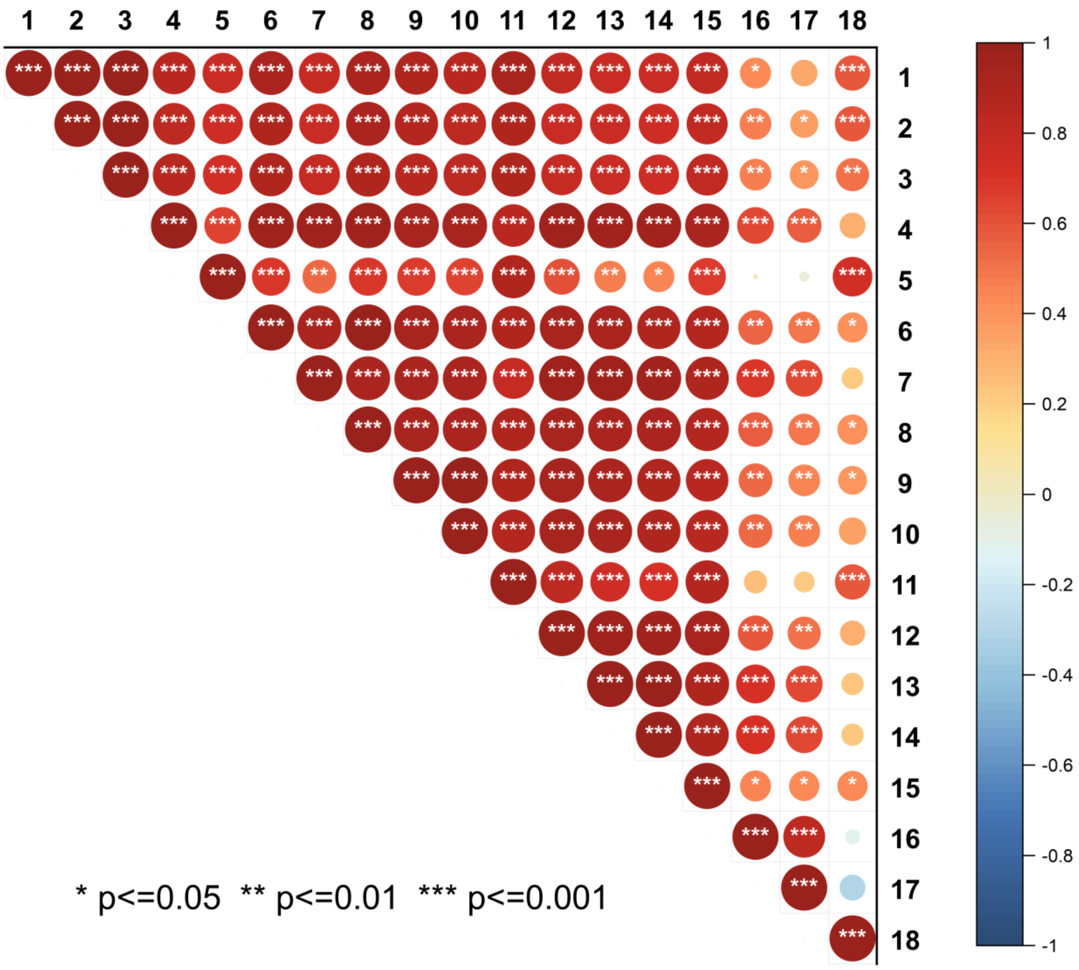

Figure S6 Correlation analysis of 18 socio-economic impact factors in influencing the number of KESUs. (1) Total Volume; (2) Annual Average; (3) Annual Average Number of Manufacturing Enterprises; (4) Regional GDP (¥ billion); (5) Primary Industry Value Added (¥ billion); (6) Secondary Industry Value Added (¥ billion); (7) Tertiary Industry Value Added (¥ billion); (8) Industrial Value Added (RMB 100 million); (9) Number of Legal Representatives; (10) Number of Corporate Legal Entities; (11) Year-End Permanent Resident Population (10,000); (12) Urban Employed Population (10,000); (13) Local General Budget Revenue (RMB 100 million); (14) Local Fiscal Tax Revenue (RMB 100 million); (15) Local General Budget Expenditure (RMB 100 million); (16) Total Resident Disposable Income (RMB); (17) Urban Resident Disposable Income (RMB); (18) Total Grain Output (10,000 tons).

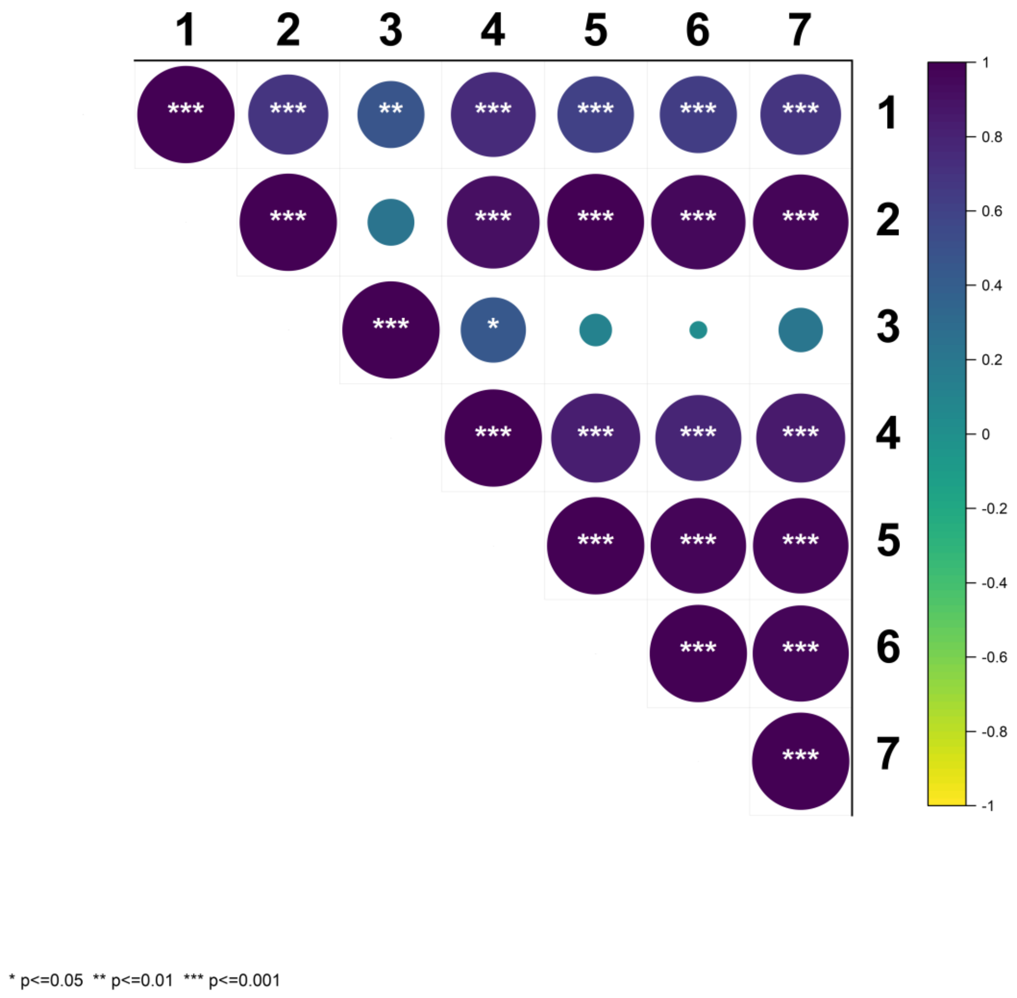

Figure S7 Correlation analysis of 7 socio-economic impact factors in influencing the number of KESUs in the city scale. (1) Number of Urban Units ; (2) Regional Gross Domestic Product (RMB billion); (3) Primary Industry Value Added (RMB billion); (4) Secondary Industry Value Added (RMB billion); (5) Tertiary Industry Value Added (RMB billion); (6) Local Fiscal Budget Revenue; (7) Local Fiscal Budget Expenditure.

Table S1 Related websites of resources for the different KESUs.

No.	Regions	Website
1	BJ	https://sthjj.beijing.gov.cn/
2	TJ	https://sthj.tj.gov.cn/
3	HE	https://hbepb.hebei.gov.cn/
4	SX	https://sthjt.shanxi.gov.cn/
5	IM	https://sthjt.nmg.gov.cn/
6	LN	https://sthj.ln.gov.cn/
7	JL	https://sthjt.jl.gov.cn/
8	HL	http://sthj.hlj.gov.cn/
9	SH	https://sthj.sh.gov.cn/
10	JS	https://sthjt.jiangsu.gov.cn/
11	ZJ	https://sthjt.zj.gov.cn/
12	AH	https://sthjt.ah.gov.cn/
13	FJ	https://sthjt.fujian.gov.cn/
14	JX	https://sthjt.jiangxi.gov.cn/
15	SD	http://sthj.shandong.gov.cn/
16	HA	https://sthjt.henan.gov.cn/
17	HB	https://sthjt.hubei.gov.cn/
18	HN	https://sthjt.hunan.gov.cn/
19	GD	https://gdee.gd.gov.cn/
20	GX	http://sthjt.gxzf.gov.cn/
21	HI	https://hnsthb.hainan.gov.cn/
22	CQ	https://sthjj.cq.gov.cn/
23	SC	https://sthjt.sc.gov.cn/
24	GZ	https://sthj.guizhou.gov.cn/
25	YN	https://sthjt.yn.gov.cn/index.html
26	TB	https://ee.xizang.gov.cn/
27	SN	https://sthjt.shaanxi.gov.cn/
28	GS	https://sthj.gansu.gov.cn/
29	QH	https://sthjt.qinghai.gov.cn/
30	NX	https://sthjt.nx.gov.cn/
31	XJ	https://sthjt.xinjiang.gov.cn/

Reference

1. Ministry of Ecology and Environment. Administrative Measures for the List of Key Environmental Supervision Units. Order No. 27 of the Ministry of Ecology and Environment, Ministry of Ecology and Environment, Beijing, P.R. China. 2023.
2. The China National Institute of Standardization. Industrial classification for national economic activities (GB/T 4754-2017). Beijing, P.R. China (2017).
